# Combination of Id2 Knockdown Whole Tumor Cells and Checkpoint Blockade: A Potent Vaccine Strategy in a Mouse Neuroblastoma Model

**DOI:** 10.1371/journal.pone.0129237

**Published:** 2015-06-16

**Authors:** Lina Chakrabarti, Clifford Morgan, Anthony D. Sandler

**Affiliations:** The Joseph E. Robert Jr. Center for Surgical Care and The Sheikh Zayed Institute for Pediatric Surgical Innovation, Children’s National Medical Center, George Washington University, Washington, District of Columbia, United States of America; Ohio State University, UNITED STATES

## Abstract

Tumor vaccines have held much promise, but to date have demonstrated little clinical success. This lack of success is conceivably due to poor tumor antigen presentation combined with immuno-suppressive mechanisms exploited by the tumor itself. Knock down of Inhibitor of differentiation protein 2 (Id2-kd) in mouse neuroblastoma whole tumor cells rendered these cells immunogenic. Id2-kd neuroblastoma (Neuro2a) cells (Id2-kd N2a) failed to grow in most immune competent mice and these mice subsequently developed immunity against further wild-type Neuro2a tumor cell challenge. Id2-kd N2a cells grew aggressively in immune-compromised hosts, thereby establishing the immunogenicity of these cells. Therapeutic vaccination with Id2-kd N2a cells alone suppressed tumor growth even in established neuroblastoma tumors and when used in combination with immune checkpoint blockade eradicated large established tumors. Mechanistically, immune cell depletion studies demonstrated that while CD8+ T cells are critical for antitumor immunity, CD4+ T cells are also required to induce a sustained long-lasting helper effect. An increase in number of CD8+ T-cells and enhanced production of interferon gamma (IFNγ) was observed in tumor antigen stimulated splenocytes of vaccinated mice. More importantly, a massive influx of cytotoxic CD8+ T-cells infiltrated the shrinking tumor following combined immunotherapy. These findings show that down regulation of Id2 induced tumor cell immunity and in combination with checkpoint blockade produced a novel, potent, T-cell mediated tumor vaccine strategy.

## Introduction

Neuroblastoma accounts for 6% of all childhood cancers in the United States, with about 700 children younger than 15 diagnosed each year. It is the third most common tumor in childhood and the most common cancer in babies younger than one. High-risk patients with unfavorable tumors continue to have dismal prognosis despite aggressive multi-modal treatment strategies [1–4]. To date, cancer vaccines have held much promise for therapy [5,6] but little clinical success. Active immunity against high-risk neuroblastoma is difficult to demonstrate, primarily due to large tumor bulk, rapid cellular proliferation and high-dose chemotherapy that weaken the patient’s immune system. In addition, neuroblastoma builds a sophisticated immunosuppressive microenvironment that prevents the development of effective T-cell immunity [7–12]. Thus, the task of establishing an effective anti-tumor response in neuroblastoma is daunting, considering the low immunogenicity of this high-risk tumor [13] along with tumor-induced immune suppression [14] and evasion.

Using a mouse model of neuroblastoma we have described a novel paradigm in tumor biology known as reversible adaptive plasticity [15] (RAP).RAP allows tumor cells to reversibly transition between highly proliferative anchorage dependent and slow growing anoikis resistant or anchorage independent phenotypes. This phenotypic heterogeneity is observed in mouse and human neuroblastoma, as well as in many other high-risk tumor types suggesting that RAP occurs during tumor growth and adaptation. A critical characteristic of RAP in mouse neuroblastoma is the necessary and abundant expression of inhibitor of differentiation protein 2 (Id2) in its anchorage dependent phenotype [16]. This is true for human neuroblastoma as well, in which we have described abundant Id protein expression. Of interest, Id proteins can be reactivated in human cancer and it is proposed that deregulated Id signaling may promote multiple attributes of malignant behavior [17]. The excessively high expression of Id in anchorage dependent neuroblastoma cells and its function as an effector of n-myc make it an important target in neuroblastoma [18,19]. To understand the role of Id2 in neuroblastoma cell plasticity, we targeted Id2 expression in Neuro2a cells with lentiviral vectors expressing Id2shRNA and found that Id2 is the key molecule modulating phenotypic transition in neuroblastoma [16].

In an attempt to determine the effect of knockdown of Id2 protein on tumorigenicity in vivo, we implanted Id2 knock down Neuro2a (Id2-kdN2a) cells in mice. Unexpectedly, most of the mice rejected the tumor cells, and subsequently were protected against further wild-type tumor cell challenge. In contrast, when immune-deficient mice were challenged with Id2-kdN2a cells the tumors grew aggressively. These findings show that down regulation of Id2 not only attenuates tumorigenicity of the neuroblastoma cells, but also renders the cells immunogenic and induced host immunity.

Immunomodulatory antibodies that directly enhance the function of T-cells potentially offer a means of overcoming immune escape mechanisms by generating effective antitumor immunity [20–22]. In particular, mouse tumor models demonstrate that blockade of the checkpoint protein, cytotoxic T lymphocyte antigen-4 (CTLA-4), a negative regulator of T cell responses, augments immunity to tumor cells when used on its own or in combination with other therapeutic interventions [6,23–25]. The combination of anti-CTLA-4 immunotherapy with agents that prime immune response is illustrated in multiple tumor models and highlights the importance of immune priming for successful anti-CTLA-4 immunotherapy. Synergistic effects of anti-CTLA-4 antibodies are demonstrated in combination with vaccines in EL4 lymphoma [26], B16 melanoma [27], prostate cancer [28] and SM1 mammary carcinoma [29] models suggesting that combination immunotherapy activates the immune system, sustains a functional response and reverses tumor tolerance. Two antibodies that block CTLA-4, ipilimumab and tremelimumab, have been evaluated in clinical trials. Ipilimumab is shown to significantly prolong the overall survival of patients with metastatic melanoma [30,31] and is approved by the United States Food and Drug Administration for the treatment of advanced melanoma. Phase II trials of these blocking antibodies are underway in adult patients with metastatic colorectal, gastric and esophageal cancers and in non-small cell lung cancer [32,33]. With the exception of a recent report suggesting CTLA-4 antibody combined with peptide antigen as an effective immunotherapy against neuroblastoma [25], there is no preclinical data on the role of immune-modulators in the treatment of neuroblastoma.

By exploiting the serendipitous finding that Id2-Kd N2a cells were immunogenic, we vaccinated mice with Id2-Kd N2a cells in combination with anti-CTLA-4 antibody. This combination vaccine strategy induced eradication of large established neuroblastoma tumors. Moreover, immune cell depletion studies demonstrated that while CD8+ T-cells are absolutely necessary for antitumor immunity, CD4+ T-cells are also required to induce a sustained long-lasting helper effect. Finally, highly functional cytotoxic CD8+ T-cells infiltrated the shrinking tumor microenvironment validating the role of T-cell immunity in this vaccine strategy. These findings should enable translation into a therapeutic patient-specific vaccine for resistant high-risk neuroblastoma tumors.

## Materials and Methods

### Animals

Female A/J, SCID and nude mice (6 weeks old) were purchased from Jackson Laboratory (Bar Harbor, ME). The animals were acclimated for 4–5 days prior to tumor challenge. All procedures were approved by the Institutional Animal Care and Use Committee of Children’s National Medical Center, Washington DC.

### Cells

Neuro2a is the murine neuroblastoma cell line derived from AJ mice and purchased from ATCC (Manassas, VA). Cells were cultured as anchorage dependent in DMEM (Gibco, Carlsbad, CA) containing 10% fetal bovine serum (FBS, Gibco) and 1% penicillin/streptomycin (Sigma, St. Louis, MO). The aggressive subclone of Neuro2a (AgN2a) cells was produced by repeated *in vivo* passaging of the cells as described previously [34].

### Human neuroblastoma samples

De-identified fresh human neuroblastoma samples were obtained from the Pathology department of CNMC. Written informed consents were obtained from the parents or guardians of the patients in accordance with the Declaration of Helsinki. All procedures involving the use of human tumor specimens were approved by the Institutional Review Board of CNMC.

### Cell transductions

The anchorage dependent Neuro2a cells were transduced with Id2-shRNA expressing lentiviral particles containing a Puromycin resistance gene (Santa Cruz Biotechnology, Santa Cruz, CA) for stable knockdown of Id2. The stable clones expressing the Id2-shRNA (Id2kd N2a) were selected using Puromycin according to the manufacturer’s instructions. Scrambled shRNA lentiviral particles were used as control and effective transfection of scrambled sh-RNA again was proven with Puromycin selection similar to the Id2-kd clones. Untransfected controls did not survive the Puromycin challenge. The knock down of Id2 was validated by western blot analysis. Luciferase expressing Neuro2a cells were constructed by transducing the Neuro2a cells with luciferase (firefly) expressing lentiviral particles (GenTarget Inc, San Diego, CA) and selecting the clones with Puromycin. Luciferase expression was determined by measuring bioluminescence in a luminometer using the Luciferase Assay System (Promega, Madison, WI).

### Antibodies

Anti-CTLA-4 antibody (9D9) and mouse IgG2b isotype control were purchased from BioXCell. Anti-rabbit Id2 antibody was purchased from Santa Cruz Biotechnology. Mouse anti-CD4-APC, CD8α-PercP, CD45-FITC, purified mouse anti-CD8α, CD4, NK1.1 and CD3 antibodies and mouse regulatory T cell staining kits were purchased from eBioscience (San Diego, CA).

### Mouse neuroblastoma therapy models

The right flank of A/J mice were injected subcutaneously (s.c) with 1x10^6^ freshly prepared tumor (Neuro2a or AgN2a) cells in 100 μl PBS on day 0. One millionId2-kd N2a cells were injected (s.c.) on their left flank on day 6 and again on day 13. The mice developed 5 mm size tumors on their right flank by day 6. Anti-CTLA-4 antibody (150 μg per mouse) or equivalent amount of IgG2b isotype control were administered intra-peritoneally (i.p.) on days 6, 9 and 12. Mice were monitored daily following tumor inoculation. Tumor growth was recorded on alternate days by measuring the diameter in two dimensions using a caliper and by imaging the mice for tumor bioluminescence using IVIS Lumina III (Perkin Elmer).Tumor volume was calculated using the formula: (large diameter x small diameter)^2^ x 0.52. A tumor size of 20 mm diameter in any dimension was designated as the endpoint and mice were euthanized at that time. Euthanasia was achieved through cervical dislocation after CO_2_ narcosis. If the tumor impaired mobility of the animal, became ulcerated or appeared infected, or the mice displayed signs of “sick mouse posture” the mice were euthanized and removed from the study group. Food was provided on the cage floor when the tumor size reached 15 mm in diameter. All the procedures are approved by the IACUC at CNMC and are in accordance with the humane care of research animals.

### *In vivo* T cell depletion models

CD8/CD4/natural killer (NK) cells were depleted by i.p. administration of purified anti-CD8α (100μg /mouse), CD4 (100μg /mouse) and NK1.1 (300μg /mouse) depletion antibodies starting a day prior to Neuro2a cell inoculation and by repeating injections on days 3, 7, and 11 after inoculation. Depletion of CD8+, CD4+ and NK1.1+ T-cells were validated using peripheral blood and analyzed by flow cytometry (>95% depletion). All the mice in depletion studies were subjected to Id2-kd N2a cell vaccination and CTLA-4 blockade as described above.

### Tumor digestion

Mouse and human tumors were weighed, and minced in 2mL of serum free RPMI. Minced tumors were placed in a 50mL tube, and filled to a final volume of 5mL serum free RPMI per 1g of tumor. Enzymatic digestion of tumors were performed with Collagenase I (Sigma), Dispase II (Roche) and DNase I (Roche) used at a final concentration of 1500U/mL, 4.8mg/mL, and 3000U/mL, respectively. The tumors were digested in a 37°C shaker bath for 20 minutes, and then placed on ice for 1–2 minutes, allowing the remaining undigested pellet to settle. The supernatant containing the single cell suspension was passed through a 40μm strainer, before being centrifuged at 250 x g for 5 minutes, and finally resuspended at the desired volume.

### Flow cytometry

Cells from mouse and human tumor digests and splenocytes were stained for CD4, CD8 and CD45 using fluorochrome conjugated antibodies described above and flow cytometry was performed in a FACSCalibur (BD Biosciences, San Jose, CA). Data analysis was done using FlowJo software (Tree Star, Inc., Ashland, OR).

### Interferon-γ by ELISA

Spleen cells were harvested and 2x10^5^cells were plated per well into 96-well round-bottom plates. The spleen lymphocytes were stimulated with the following: 2x10^4^ wild type Neuro2a cells, 2x10^4^ Id2 knock down Neuro2a cells and 1.0 μg/mL of purified anti-CD3. Splenocytes were then incubated at 37°C for 48 hours prior to IFN-γ assay. Cell culture supernatants were collected from triplicate wells after stimulation and IFN-γ secreted by lymphocytes were measured by ELISA using purified capture and biotinylated detection antibody pairs (BD Biosciences). The ELISA plates were read using the EnSpire 2300 Multilabel Reader (Perkin Elmer, Shelton, CT) at 450 nm.

### Chromium-51 release cytotoxicity assay

Cytotoxic T lymphocyte activity of tumor infiltrating lymphocytes was determined by standard ^51^Cr release assays. In brief, Neuro2a cells (target) were incubated with 0.2 mCi Na[^51^]CrO_4_ for 45 min at 37°C. Cells were washed twice with complete medium and transferred to round-bottom 96-well plates at 5 × 10^3^ cells/well. CD8+ T-cells (effector) were purified from the unvaccinated growing and vaccinated shrinking total tumor digest using the MACS cell separation system (Miltenyi Biotec, Auburn, CA). Due to low abundance of the infiltrating lymphocytes in the growing tumors, purified T-cells were pooled together from four unvaccinated growing tumors for the assay. Effector T-cells were added to target tumor cells at varying numbers in a final volume of 0.2 ml to give the effector:target ratios as indicated in the Figure legends. After 4 hours incubation at 37°C, 0.1 ml of supernatant was harvested, and released radiolabel was determined by scintillation counting. Maximal release from targets was determined by treatment of cells with 1% Triton X-100, spontaneous release was determined from cultures of labeled targets incubated with medium only, and the formula used for determination of specific lysis was: [(experimental release − spontaneous release)/(maximal release − spontaneous release)] × 100.

### Statistical analysis

The two-tailed Student’s t-test was used to determine statistical significance between groups unless otherwise stated. A probability level of p<0.05 was considered to be statistically significant.

## Results

### Knock down of inhibitor of differentiation protein 2 (Id2) attenuates neuroblastoma tumor cells and induces host immunity

**Fig 1:**
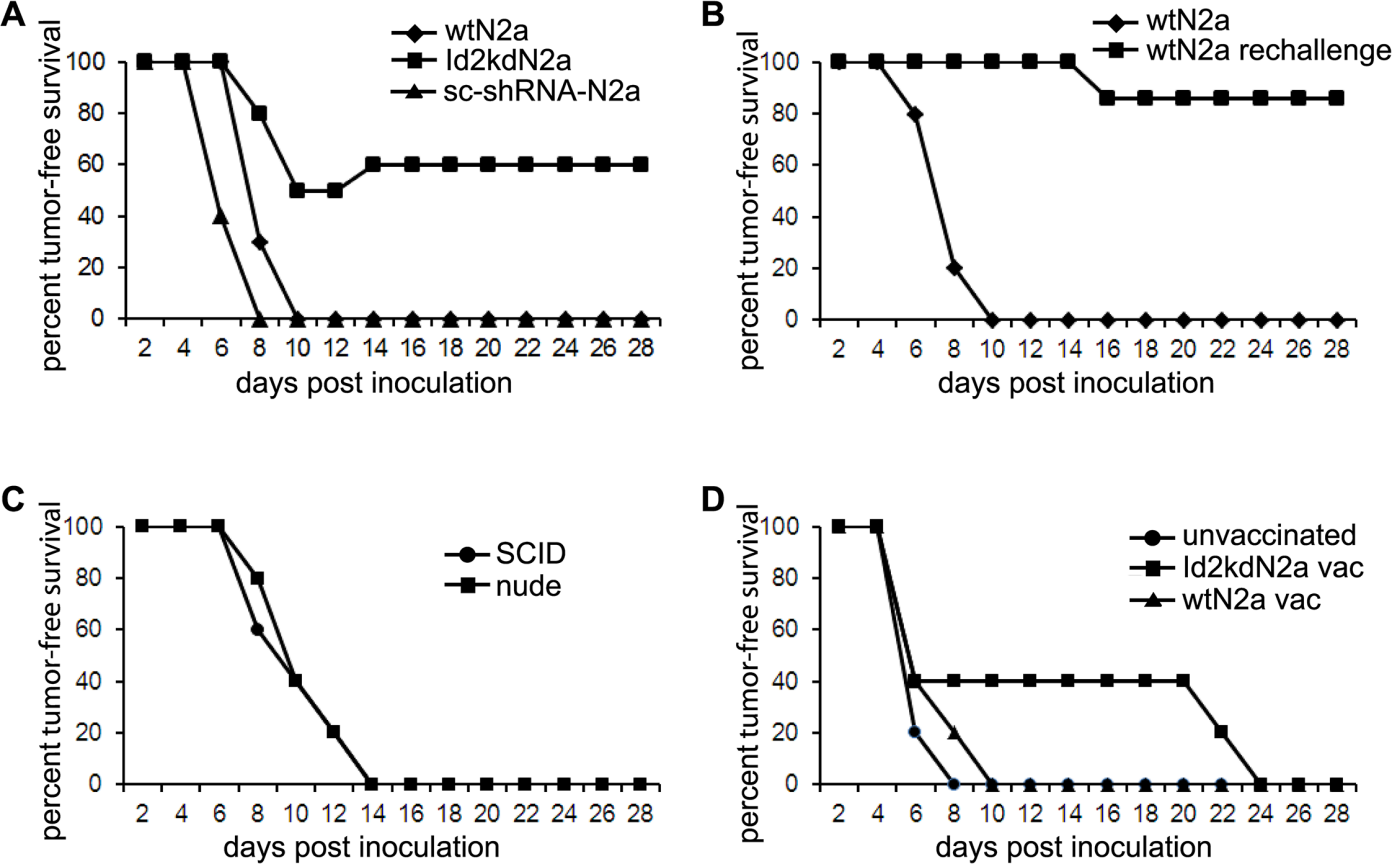
Id2 knock down attenuates tumorigenicity and induces host immunity. (**A**) Sixty percent of mice (n = 9/15) challenged (right leg) with Id2 knock down Neuro2a (Id2kdN2a) cells rejected tumor and survived tumor-free, whereas all mice challenged with either wild type Neuro2a (wtN2a, n = 20) or scrambled shRNA lentivirus transfected Neuro2a (sc-shRNA-N2a, n = 5)) cells died from tumor burden. (**B**) Tumor free survivors from (A) were re-challenged with wtN2a cells into their left leg 6 weeks after they cleared the tumor and 8 out of 9 mice were protected from tumor growth. (**C**) SCID and nude mice grew tumors aggressively following inoculation with Id2kdN2a cells. (**D**) Following inoculation of wtN2a cells in the right leg, Id2kdN2a (n = 10) and wtN2a (n = 5) cells were vaccinated into the left leg of mice, 3 and 5 days later respectively. The wild type tumor growth on the right leg was delayed in Id2kdN2a vaccinated mice when compared to control unvaccinated mice or wtN2a vaccinated mice.

**Fig 2:**
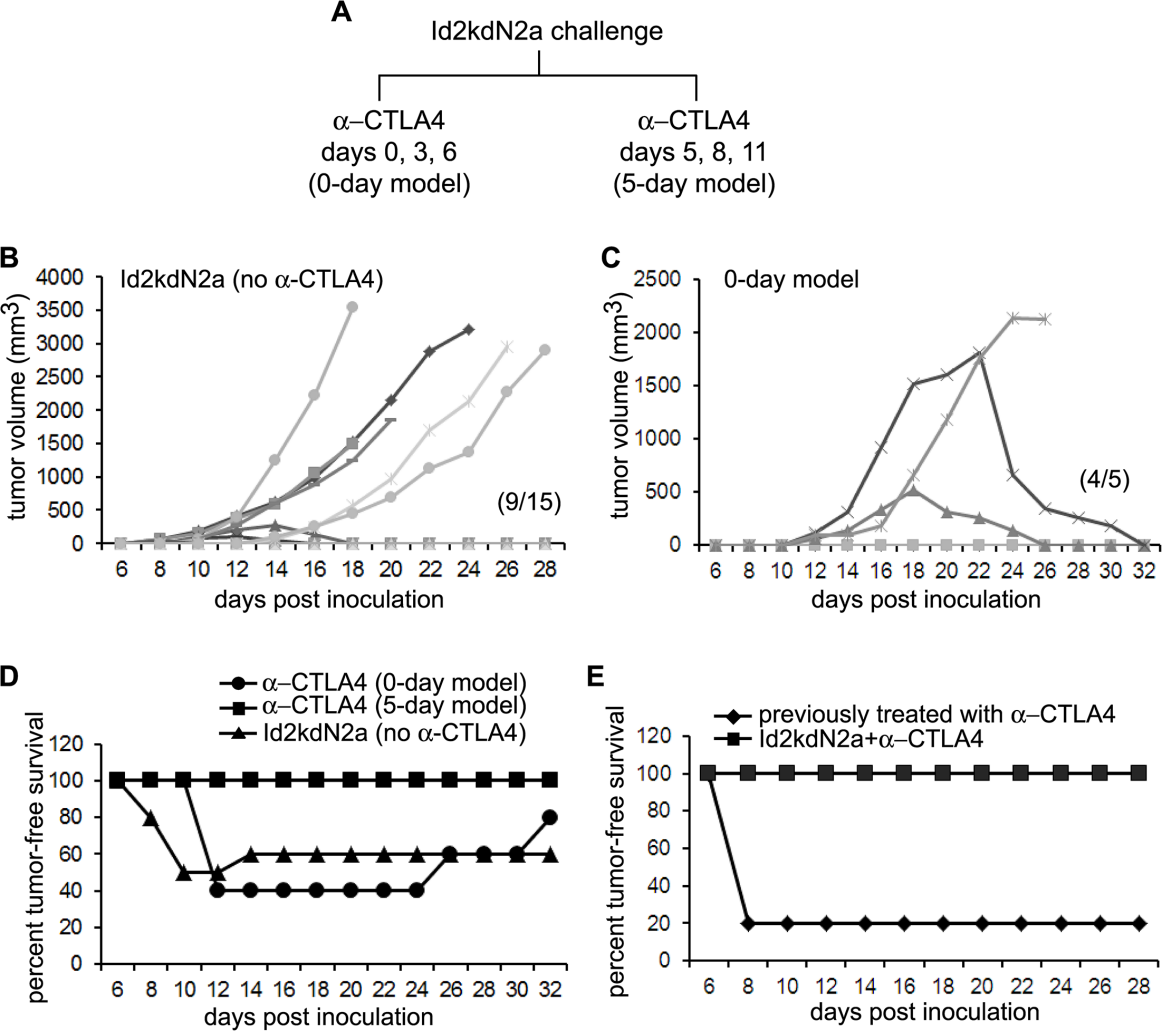
CTLA-4antibody enhances anti-tumor effect of Id2-kd N2a cell vaccine in prophylactic tumor models. (**A**) Schematic diagram of the prophylactic Neuro2a tumor models. Two models (0-day and 5-day) were tested, in which three doses of α-CTLA4 antibody were administered either days 0,3,6 or 5,8,11 following Id2kdN2a cell inoculation into the right leg of the mice (n = 5 for each model). (**B, C**) Tumor growth curves show that 60% of mice challenged with only Id2-kd N2a cells (no α-CTLA4) survived tumor free (B) and in the 0-day model (C) 40% of mice never grew tumor and another 40% cleared the tumor slowly and became tumor free. Graph depicts tumor growth in individual mice. The parenthesis indicates number of mice that survived tumor free. (**D**) All mice in the 5-day model survived tumor free, in contrast to 80% in the 0-day model or 60% in the Id2kd cells model. (**E**) Six weeks after tumor clearance, the tumor-free mice from (B) (n = 9) were re-challenged with wtN2a cells into their left leg and 100% were completely immunized against wild type tumor growth. Only 1 of 5 mice (20%) treated previously with anti-CTLA4 antibody alone survived tumor free after wtN2a challenge at 6 weeks.

We recently described that targeting Id2 expression in anchorage dependent Neuro2a cells reduced their proliferation, increased the rate of tumorsphere formation and activated tyrosine kinase and TGFβ signaling pathways [16]. In an attempt to determine the effect of Id2 down- regulation on Neuro2a tumorigenicity and adaptive transition *in vivo*, we knocked down Id2 with lentiviral vectors expressing Id2 shRNA in neuro2a cells. The knock down was confirmed by western blot analysis of Id2 protein expression [16]. Implantation of Id2-kd N2a cells surprisingly resulted in tumor rejection in 60% of mice (Figs 1A and 2B) and these mice were subsequently protected against further wild-type Neuro2a tumor cell challenge (Fig 1B). In contrast, severe combined immune-deficient (SCID) and nude mice grew Id2-kd tumors aggressively (Fig 1C), highlighting the immunogenicity of the Id2-kd cells. The tumorigenicity of scrambled shRNA lentivirus transfected Neuro2a cells (sc-shRNA-N2a) was comparable to the wild-type cells (Fig 1A). These findings suggest that down regulation of Id2 not only attenuates tumorigenicity in the mouse model, but also induced host immunity indicating the potential role of Id2-kd N2a cells as an antigenic vehicle for an attenuated, whole tumor cell vaccine.

To this end, we tested the use of Id2-kd N2a cells in a therapeutic treatment model of established neuroblastoma tumor. Id2-kd N2a cells were vaccinated into the left leg of mice, three days following inoculation of wild type Neuro2a tumor cells in the right leg, and tumor growth was monitored. None of the mice developed tumor at the site of Id2-kd N2a cell injection, while the growth of wild type tumor on the opposite leg was delayed when compared to control unvaccinated mice (Fig 1D). In contrast, when wild type cells were injected into both hind legs 5 days apart, tumors grew aggressively on both sides, suggesting the absence of concomitant immunity in the wild type Neuro2a, validating the immunogenicity and potential use of Id2-kd N2a cells as a tumor vaccine.

### CTLA-4 blockade enhances anti-tumor immunity induced by Id2 knock down neuroblastoma cells

In order to determine if the therapeutic effect of the Id2-kd N2a whole tumor cell vaccine could be enhanced, we elected to combine this therapy with immune checkpoint blockade in the form of anti-cytotoxic T lymphocyte associated antigen 4 (CTLA-4) antibody. Indeed, in a prophylactic tumor vaccination model (Fig 2A) we observed that CTLA-4 blockade started on day 5 after Id2-kd N2a cell implantation resulted in 100% tumor rejection (Fig 2D) in contrast to 80% when given on the same day as the Id2-kd N2a cells (Fig 2C and 2D) highlighting the synergy between immune priming and immune modulation that is required for effective tumor vaccine therapy. Moreover, all tumor-rejected mice developed complete immunity against wild type tumor cells challenged 6 weeks after tumor clearance (Fig 2E).

**Fig 3:**
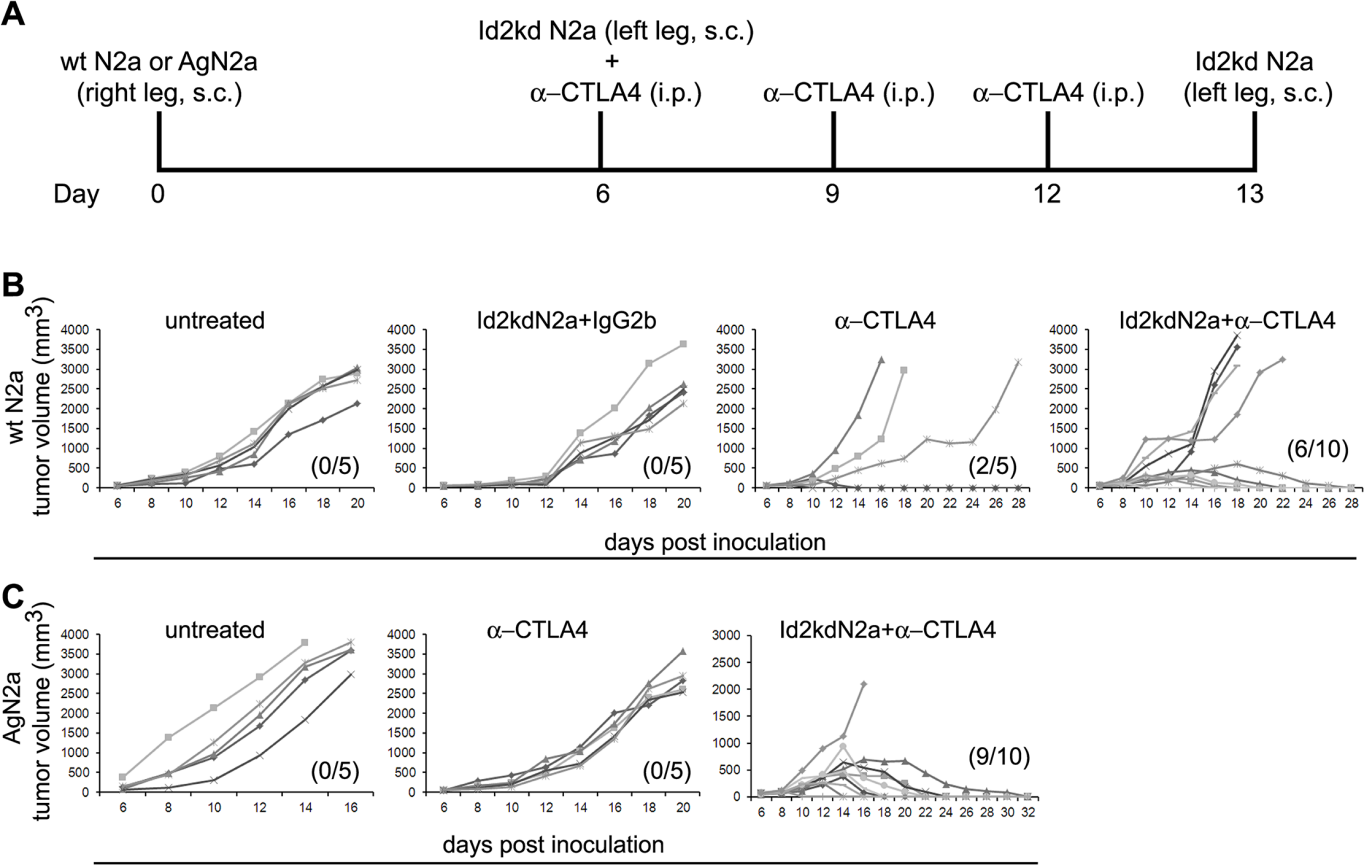
Combination of Id2kd N2a and α-CTLA4 antibody as a therapeutic vaccine. (**A**) Schematic diagram of the therapeutic vaccine strategy. Two established tumor models, namely Neuro2a (wtN2a) and AgN2a were tested, where mice challenged with either wtN2a or AgN2a cells were subjected to a combination immunotherapy with Id2kdN2a and α-CTLA4 antibody starting at day 6 after inoculation. Neuroblastoma tumors are normally visible (5mm in diameter) in AJ mice by day 6. Tumor growth curves in individual mice of wtN2a (**B**) and AgN2a (**C**) cells show that the Id2kd tumor cell vaccination combined with immune-modulation cures mice with established tumor. Parentheses indicate the number of mice that survived tumor free.

**Fig 4:**
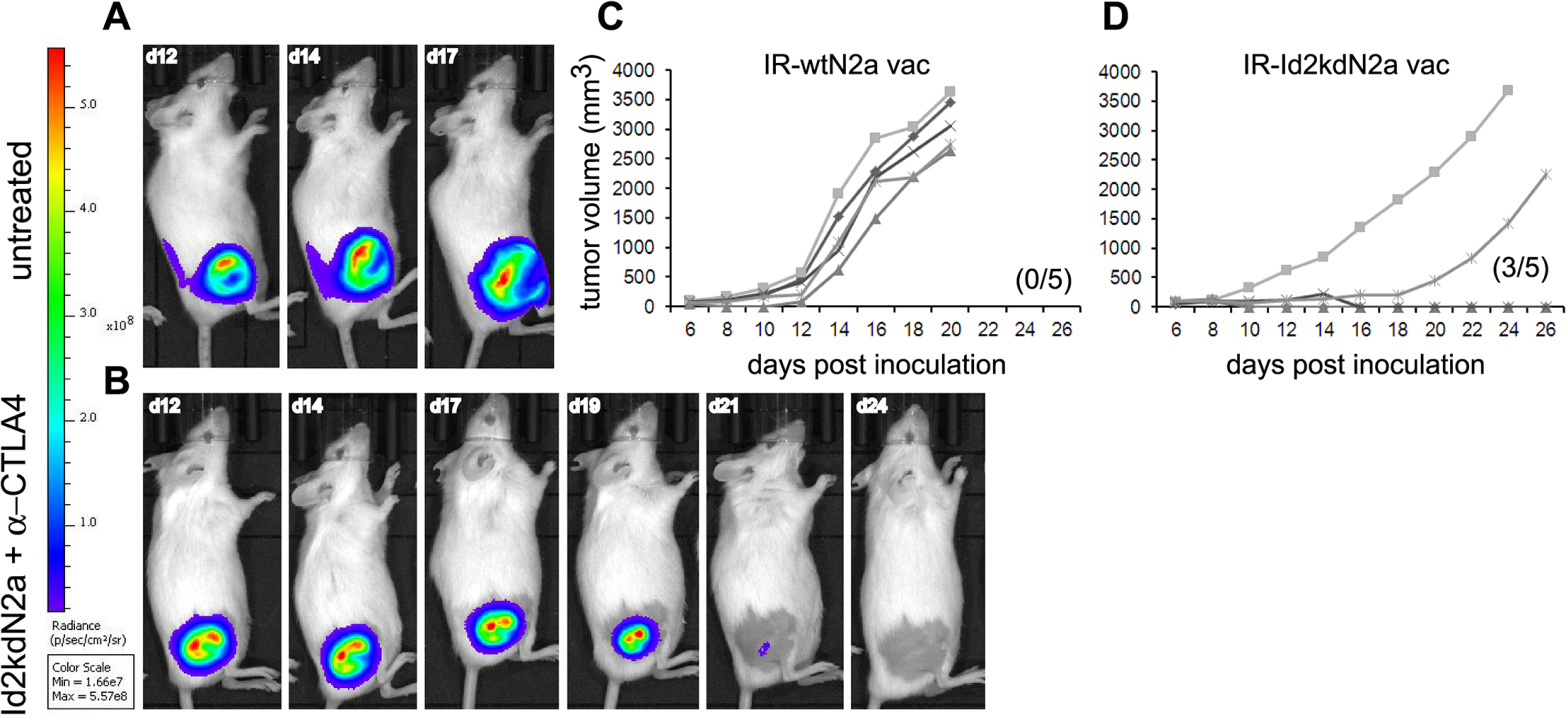
Tumor clearance and effect of irradiation on tumor cell immunogenicity. (**A**, **B**) Representative mouse from untreated and combination therapy group showing growing and shrinking tumor respectively. All bio-luminescent images were analyzed under the same scale.(**C**, **D**) Irradiated Id2 knock down Neuro2a (IR-Id2kdN2a, n = 5) and wild type Neuro2a (IR-wtN2a, n = 5) were compared as whole tumor cell vaccine antigen source in combination with α-CTLA-4 antibody against AgN2a (see Fig 3A). IR-Id2kdN2a vaccine was able to eradicate 60% of tumors in comparison to IR-wtN2a cells which had no effect on growth of the AgN2a tumors. All mice in (C) were sacrificed on day 20 due to large tumor burden. The parenthesis indicates number of mice that survived tumor free.

Encouraged by the effectiveness of this prophylactic model, we reasoned that the combination of the Id2-kd N2a vaccine platform with anti-CTLA-4 antibody may break tolerance even in the presence of large established tumors and induce potent T-cell immunity. We thus examined the combined vaccine/immunotherapy effect in an established neuroblastoma tumor model (Fig 3A). Combined therapy eradicated 60% of established Neuro2a tumors in the wild-type model (Fig 3B) and 90% in AgN2a (aggressive non-immunogenic) model (Figs 3C, 4A and 4B), whereas, CTLA-4 blockade alone resulted in 40% tumor regression in the Neuro2a model and had no effect on tumor growth in the AgN2a model (Fig 3B and 3C). Of interest, again none of the mice developed tumor at the site of Id2-kd N2a cell injection in this established tumor model.

### Irradiation of Id2-kd N2a whole tumor cells does not impair immunogenicity

Live attenuated vaccines induce long-lived cellular and humoral immunity, but safety concerns limit their utilization. Inactivation and attenuation of pathogens have been strategies for vaccine development since the advent of vaccination [35]. Therefore, in lieu of the potential risk of live albeit attenuated Id2-kd N2a tumor cells we sought to determine whether irradiation of these cells would dampen the effects of this tumor cell vaccine strategy. We subsequently tested irradiated (35 Gray) Id2-kd N2a cells as a whole tumor cell vaccine antigen source in combination with anti-CTLA-4 antibody against AgN2a (Fig 3A) and found that 60% of mice eradicated established tumors (Fig 4D). In contrast, irradiated wild type Neuro2a cells administered in a similar combination fashion, had no effect on growth of the AgN2a aggressive tumor cells (Fig 4C). This observation again supports the antigenicity of Id2-kd N2a cells for vaccination, but is also encouraging in that irradiation extends the margin of vaccine safety. Although irradiation of tumor cell vaccines is known to evoke potent anti-tumor immunity in melanoma and lung cancer models [36,37], we find neither a favorable nor particularly adverse effect of irradiation on the antigenic properties of the Id2-kd cells in neuroblastoma. Taken together these results suggest that Id2-kd N2a cells provide a safe and effective source of tumor antigenicity and in combination with checkpoint blockade induce potent tumor immunity.

### CD8+ T-cells are necessary and CD4+ T-cells are required for neuroblastoma tumor eradication

**Fig 5:**
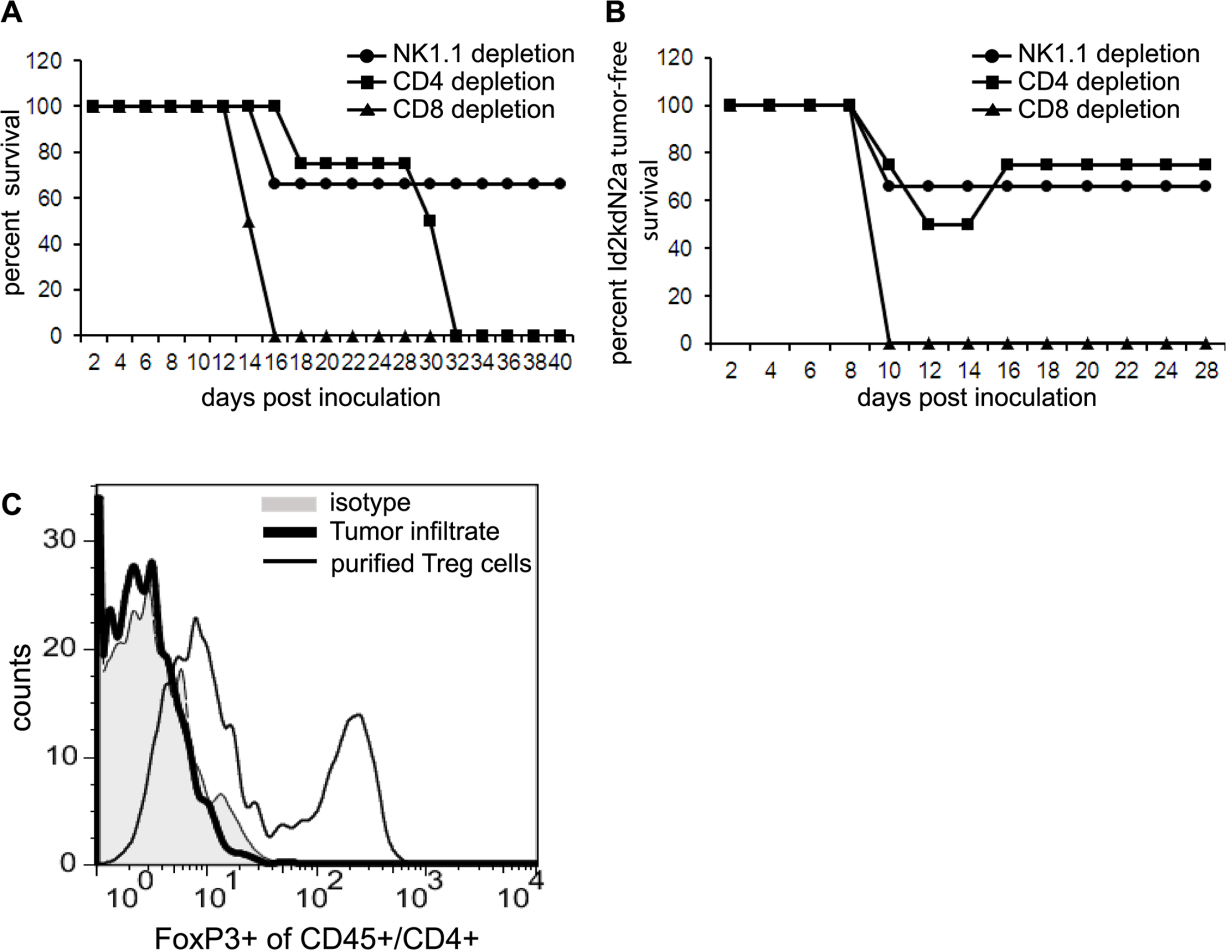
T cell immunity required for tumor eradication following combination therapy. Mice were depleted of specific T cell subsets by systemic administration of antibodies against CD4, CD8 and NK cells and subjected to combined Id2kdN2a cells and α-CTLA-4 antibody treatment strategy (see Fig 3A) (**A**) *In vivo* depletion of CD4+, CD8+ and NK+ cells (n = 5 for all groups) shows that the therapeutic effect of the combined treatment was completely abrogated by CD8+ cell depletion. Mice lacking NK cells were for the most part able to reject their tumors following therapy. CD4+ T cell depletion initially appeared to have minimal adverse effects on the therapeutic vaccine strategy, but after 4 weeks all mice in the CD4 depletion group developed delayed tumors. (**B**) All CD8+ T cell depleted mice developed tumors at the site of Id2kdN2a cell vaccination as well. (**C**) Cell infiltrates from the tumors of CD4 depleted mice were stained with anti-CD45-FITC, CD4-APC and FoxP3-PerCPCy5.5 antibodies to identify Treg cells; there was no evidence of Treg cell infiltrate in the late developing tumors from CD4 depleted mice. Purified Treg cells were used as positive control.

To explore which immune effector cells are critical for eradicating the established tumors following combined vaccine therapy, we depleted specific T cell subsets by systemic administration of antibodies against CD4, CD8 and Natural Killer (NK) cells. *In vivo* depletion of cell phenotypes was confirmed by blood sampling. Depletion of CD8+ T-cells completely abrogated the therapeutic effect of combined Id2-kd N2a cells and anti-CTLA-4 antibody (Fig 5A). Interestingly, all CD8+ T cell depleted mice also developed tumors at the site of Id2-kd N2a cell vaccination (Fig 5B), again demonstrating the importance of intact immunity. Mice lacking NK cells were for the most part able to reject their tumors following therapy (Fig 5A). CD4+ T cell depletion initially had minimal adverse effect on the therapeutic vaccine strategy and 75% of mice lacking CD4+ T-cells remained tumor free for 4 weeks. Interestingly, all mice in the CD4 depletion group eventually developed delayed tumors, after the 4 week period (Fig 5A). To determine if re-accumulation of CD4+ regulatory T-cells (Treg) cells played a role in tumor relapse, cell infiltrates from the tumors of CD4 depleted mice were stained with anti-CD45, CD4 and FoxP3 antibodies. No evidence of Treg cell infiltrate (Fig 5C) was identified; indicating that tumor relapse in CD4+ T cell depleted mice is not mediated by the late accumulation of Treg cells. The most likely explanation is that depletion of CD4+ T-cells during combined immunotherapy failed to help with activation of cytotoxic CD8+ T-cells resulting in inadequate immunity. Inadequate immunity is shown by the need of CD4 help to obtain complete activation of CD8 cytotoxic T-cells. Taken together, these results suggest that both CD4+ and CD8+ T-cells are required for immunity and effective tumor cell elimination following combination immunotherapy.

### Robust T-cell immunity mediates tumor rejection in both mouse and human neuroblastoma

**Fig 6:**
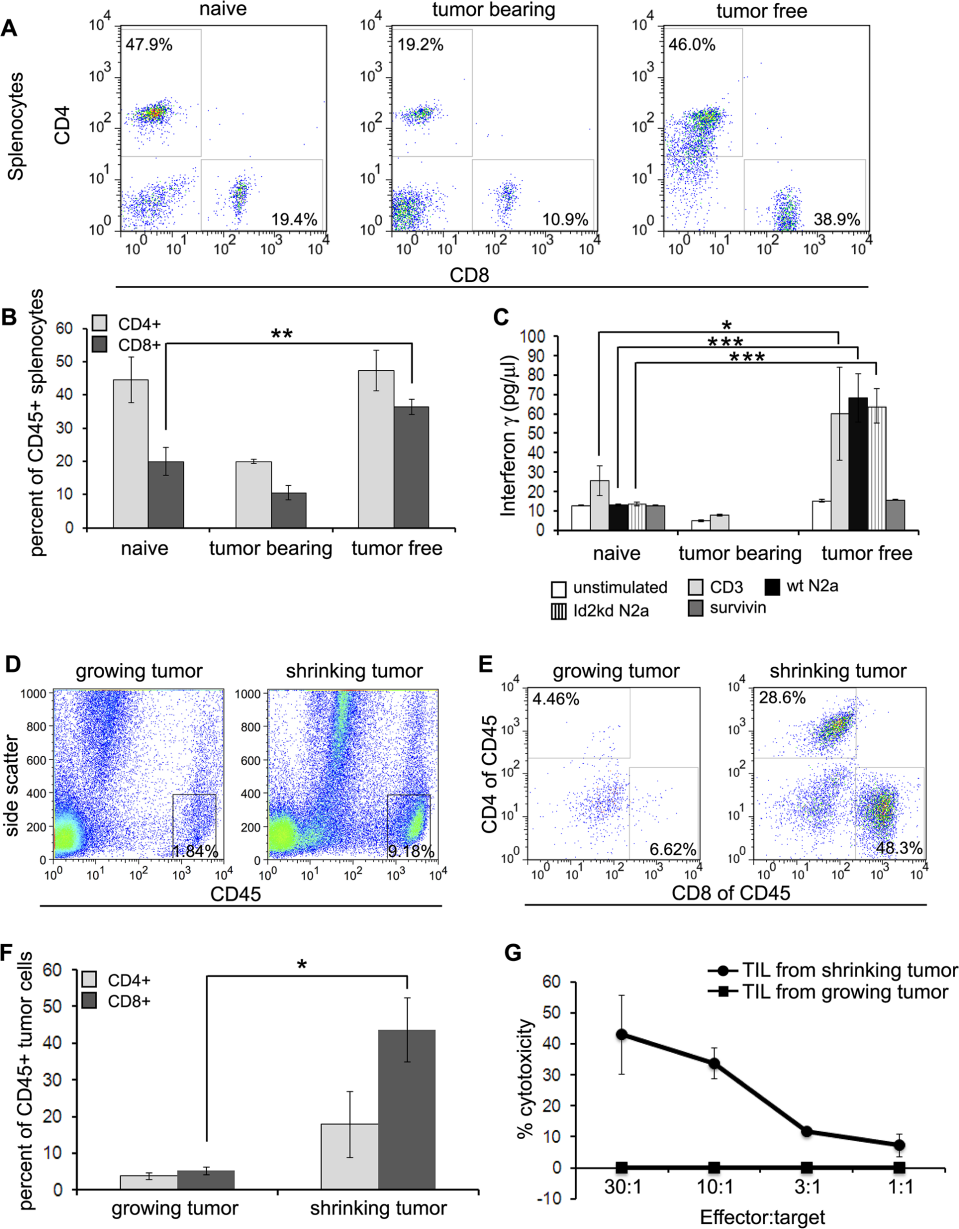
Enhanced *in vivo* immune response mediates tumor clearance. (**A**) Representative flow cytometry plots showingCD4+ and CD8+ T-cells in CD45+ splenocytes of naïve (n = 5), tumor bearing (n = 3) and tumor free (n = 5) mice. (**B**) Graphical representation of (A) indicating significant (** p = 0.013) increase in CD8+ cells in the spleens of mice cleared of tumor. (**C**) Splenocytes of mice that cleared tumor had enhanced IFNγ secretion following stimulation with CD3 (* p<0.02),wtN2a or Id2kdN2a cells (*** p<0.0001). (**D**) Remarkable increase in CD45+ cells detected in the shrinking tumor.(**E**, **F**) Tumor infiltrating lymphocytes (TIL) following vaccination were quantified and a massive infiltration of CD8+ T-cells was detected in the shrinking tumors (n = 5) as opposed to the growing tumors (* p<0.02). (**G**) Chromium^51^ release assay exhibited potent cytotoxic activity of CD8+ TIL from shrinking tumor (n = 3); whereas the TIL isolated from growing tumors (n = 4) show no activity at all. Data presented as mean ± S.D.

The prior studies clearly describe indirect evidence for T-cell immunity as a mechanism of tumor rejection following Id2-kd/checkpoint blockade therapy. We thus sought to define the *in vivo* cellular response in mice that cleared tumor following combination therapy. Splenic CD8+ T cell counts were significantly higher in mice that were vaccinated and cleared tumor, compared to naïve mice that were not vaccinated nor challenged with tumor (Fig 6A and 6B). The production of IFNγ from T-cells is a hallmark of immune cell response, thus we measured IFNγ secretion from splenocytes following antigen stimulation. The splenocytes of mice that cleared tumor secreted significant amounts of IFNγ following stimulation with wild type Neuro2a or Id2kd N2a cells (Fig 6C) compared to splenocytes taken from naïve mice.

To gain more insight into the mechanism of tumor cell rejection, we investigated the microenvironment of shrinking mouse tumors by quantifying tumor infiltrating lymphocytes (TIL) following vaccination. TIL are frequently found in tumors and are effective at delaying tumor progression suggesting the potential influence of immune cell infiltrates on patient prognosis [38–41]. We found a massive increase in CD45+ cells as well as a robust infiltration of CD8+ T-cells in the shrinking tumors following combination immunotherapy (Fig 6D–6F). To elucidate the effector function of the TIL, freshly isolated and purified tumor infiltrating CD8+ T-cells were subjected to standard chromium^51^ release assay. CD8+ TIL exhibited potent cytotoxic activity against wild type Neuro2a cells without *ex vivo* recovery (Fig 6G). This finding indicates the high functionality of these cells and further emphasizes the key role that CD8+ T-cells play in inducing effective immunity and tumor regression.

**Fig 7:**
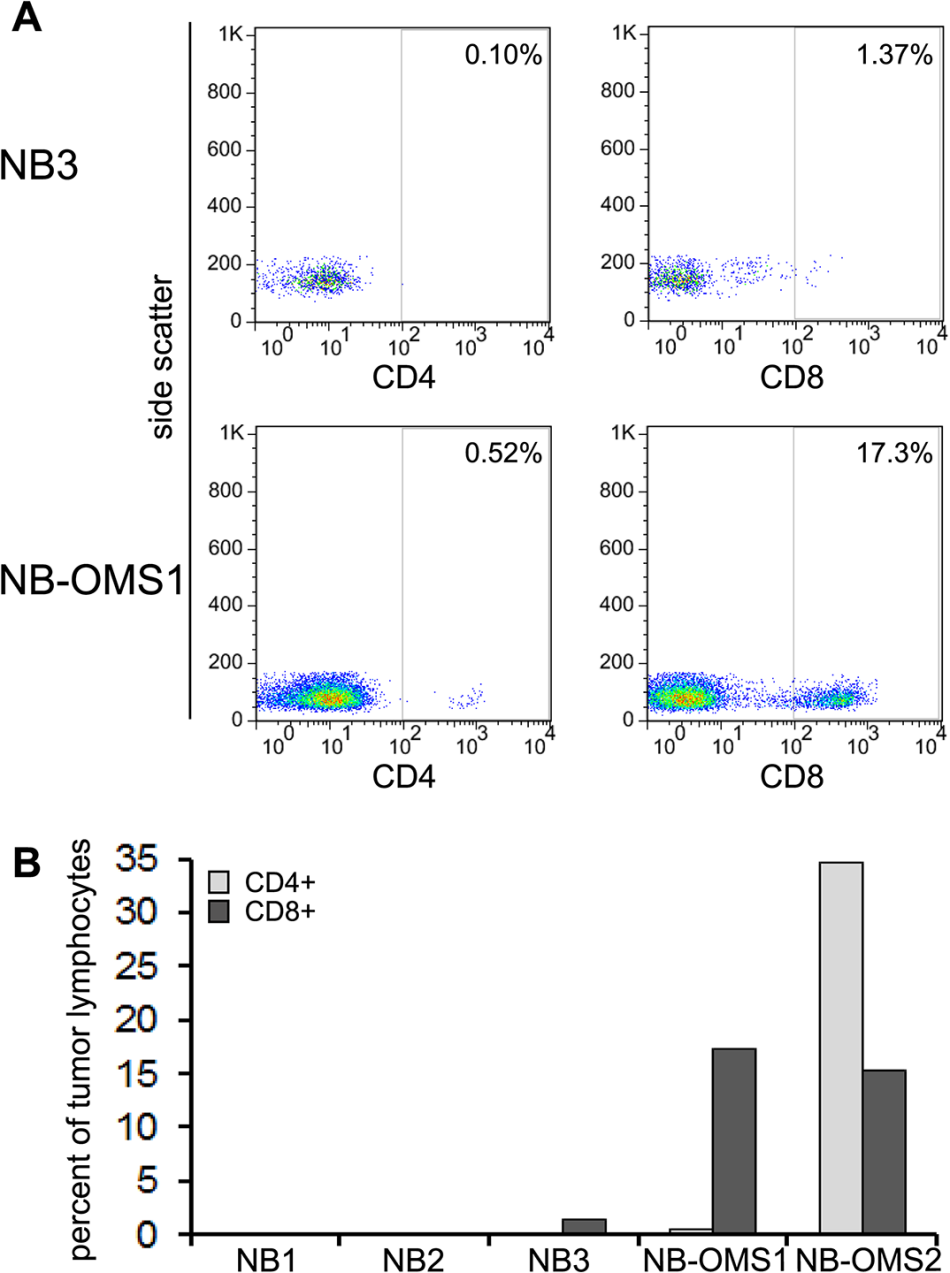
TIL in human neuroblastoma samples. Five freshly harvested human neuroblastoma specimens were analyzed for TIL; two of the tumors were from patients with Opsoclonus/myoclonus syndrome (OMS). (**A**) Representative flow cytometry plots showing CD4+ and CD8+ T-cell population in the neuroblastoma tumor lymphocytes. (**B**) Graphical representation of (A) indicating a remarkable infiltration of CD8+ T-cells in the two tumors associated with OMS (NB-OMS) as opposed to the other three neuroblastoma (NB) tumors that had minimal TIL.

As a clinical correlate of T cell immunity in neuroblastoma we examined freshly harvested human tumor specimens for TIL presence. We observed minimal CD8+ cells in several tumors with poor prognosis (0 to 1.3% of tumor lymphocytes) (n = 3), whereas in neuroblastoma tumors from patients with Opsoclonus Myoclonus Syndrome (OMS) (n = 2) a robust infiltration of CD8+ cells was noted (15.4 and 17.3% of tumor lymphocytes) (Fig 7A and 7B). It is now well documented that although childhood neuroblastoma carries a significant mortality rate, neuroblastomas associated with OMS tend to be low grade and have a significantly more favorable outcome [42–44].

## Discussion

The ability of cancer cells to escape immune surveillance is a common hallmark of cancers with poor prognosis [45]. Neuroblastoma is an example of a tumor with high-risk stratification, frequently exhibiting a poor prognosis and recurrent disease following initial therapy. Unlike several other childhood solid tumors, successful treatment of high-risk neuroblastoma remains a major challenge with limited effective therapeutic options. Strategies to circumvent treatment failures are intensively studied and amongst these strategies, immunotherapy has attracted much attention. Our current results offer a novel pre-clinical strategy combining targeted gene knock down and checkpoint blockade as a potent, effective, tumor-specific vaccine strategy for neuroblastoma. This strategy is dependent on a robust cytotoxic CD8+ T lymphocyte (CTL) response that is supported by CD4+ T helper cells for tumor eradication.

Recent advances in the understanding of mechanisms regulating T-cell activation have made significant progress in cancer immunotherapy. The activation state of the innate immune system is thought to play a critical role in either the induction of immunity or tolerance after encountering tumor antigen. In contrast to the considerable literature documenting immunological responses to melanoma in humans and in mouse models, there is a scarcity of data concerning immunological responses to neuroblastoma. Based on present concepts of immunogenicity, it comes as a surprise that live tumor cells induce an immune response at all. The whole tumor cell vaccine studied in this work, contains no pathogen associated molecular patterns nor artificial inflammatory cytokines; yet potent immunogenicity is attained by targeting a key molecule of reversible adaptive plasticity. Id2-kd N2a cells induced immunity in a syngeneic immune-competent mouse model, yet these tumor cells grew unabated in immune-compromised hosts. In the immune competent host, long-term immunity was induced against subsequent wild-type tumor challenge and of clinical interest; immunogenicity of the knock-down cells was maintained following irradiation. The mechanism of tumor cell immunogenicity in Id2-kd cells is unknown, but is clearly a perturbation in molecular homeostasis of the tumor cells. Changes induced by targeting Id2 in the tumor cell could vary from increased antigenicity, enhanced antigen presenting cell uptake or loss of immune-suppressive or immune-evasive mechanisms. Four Id proteins (Id1, Id2, Id3 and Id4) are described [46], and may function in a redundant manner. We targeted Id2 in the mouse neuroblastoma cell line, as this was the most dominant and differentially expressed protein (~20 fold) between the anchorage dependent and independent cell phenotypes [16]. In other mouse and human tumor cell lines Id2 was not dominant whereas Id1 and/or Id3 were (unpublished data). Id1 and/or Id3 may be better targets in other cancer types for inducing the attenuated, immunogenic effect observed following Id2 knock down in Neuro2a cells. The effects described with Id2 targeting may or may not be expanded to other cell lines; research to determine applicability of this whole tumor cell vaccine strategy for other tumor types is underway.

Despite the presence of tumor associated antigens, tumor growth enhances antigen specific expansion of Treg inducing tumor tolerance [47,48]. Similarly, vaccination alone can induce limited immunity with expansion of regulatory T-cells or by inhibition of T-cell activation. Treatment with monoclonal antibody specific for cytotoxic T lymphocyte associated antigen-4 (CTLA-4), a checkpoint protein expressed on T-lymphocytes has emerged as an effective cancer therapy. The effect is most likely induced by both enhancing T-cell expansion and/or by selective Treg depletion within the tumor mass [49,50].

Immune checkpoint modulation has become a clinically relevant therapy approach in melanoma and lung cancer [27,51,52]; in particular, anti-CTLA-4 antibody shows promising results in clinical trials in melanoma patients [30,31]. Like melanoma, neuroblastoma is derived from neural crest stem cells and shares common antigenic determinants [53]. A phase I clinical trial is underway for resistant tumors in children and adolescents using anti-CTLA4 antibody. Although there is clear evidence that targeting checkpoint receptors activates endogenous anti-tumor immunity with clinical benefit, the question of how checkpoint efficacy can be further improved through rational combination therapies is essential. Our current pre-clinical results provide a convincing treatment option, in which the combination of Id2kd neuroblastoma tumor cells and CTLA-4 antibody prime a functional tumor-specific T cell response, increase immune cell access to the tumor site, enhance anti-tumor immune cell function and eradicate significant tumor burden. Our data in this primary tumor model indicates a synergy between CTLA-4 blockade and Id2-kd N2a cells. Mice treated with either the altered cells or antibody alone in the non-immunogenic aggressive tumor model had either marginal or no reduction in tumor growth, whereas the combination of both resulted in significant tumor clearance. This suggests that an additional source of antigen from the cell-based vaccine contributes to T-cell priming, which is enhanced by blockade of CTLA-4 mediated inhibitory signals of T-cell activation. The ideal timing of combination vaccination and checkpoint blockade administration is uncertain, but we observed improved cell rejection when anti-CTLA-4 antibody administration lagged several days behind Id2-kd N2a cell implantation. This finding suggests that a critical window exists in which antigen processing occurs prior to checkpoint blockade.

Our results show that the vaccine’s tumor ablative effect requires both CD8+ and CD4+ T-cells. The immune cell depletion study proves that cytotoxic CD8+ T-cells (CTL) are indispensable for tumor rejection, but also demonstrates the requirement for CD4+ T cell help for effective induction of the CD8+ CTL response. Furthermore, we demonstrate a massive influx of activated CD8+ CTL in regressing mouse tumors as was also observed in two human neuroblastomas associated with Opsoclonus Myoclonus Syndrome (OMS). A large body of evidence has uncovered a correlation between the presence of lymphocyte infiltration and the survival of patients affected by many types of cancer including neuroblastoma [39,41,54,55]. In particular, presence of CD8+ T-cells was associated with a favorable prognosis [54,56] and is considered the major component of an effective immune response to most tumors. Evidence suggests children with coincident OMS and neuroblastoma have favorable outcome and become long-term survivors [42–44]. Moreover, similar to our finding, diffuse and extensive lymphocytic infiltration was observed in neuroblastic tumors associated with OMS [57] suggesting the role of immune surveillance in recognizing and eradicating unfavorable tumor cells in these patients.

The opsoclonus and myoclonus observed in this form of favorable OMS neuroblastoma is probably a secondary autoimmune response to tumor immunity. Despite the often rapid, potent and complete tumor rejection observed in our pre-clinical study, the mice remained well and showed no signs of illness in the form of potential autoimmunity. Although no specific testing was undertaken to evaluate immune related adverse events (irAEs) in the animals, they looked well and were followed for months following therapy. It is possible that the altered whole tumor cell vaccine preferentially targets tumor-specific antigens as opposed to tumor-related antigens, but this postulation is speculative as the actual antigen targets are unknown. Although it is described that the threshold of inducing auto-immunity is lower than that of producing tumor immunity [58], the well-being of the mice in this model is reassuring, but further investigation prior to clinical translation is needed.

In conclusion, the work presented demonstrates that an attenuated Id2-kd whole neuroblastoma cell vaccine is safe in mice; induces broad tumor-specific cellular immunity, protects against tumor formation in prophylactic tumor models and in combination with the clinically relevant checkpoint immune modulator CTLA-4 antibody, eradicates large established neuroblastoma tumors. This work should enable translation of our findings into a therapeutic patient-specific vaccine for resistant neuroblastoma tumors. The data also provide compelling evidence for the development of potent tumor specific vaccine strategies, based on the combination of targeted gene knock down in tumor cells and checkpoint immune-modulation for other aggressive high-risk solid tumors.
